# Cephalexin-Induced Autoimmune Hemolytic Anemia: A Case Report

**DOI:** 10.7759/cureus.102910

**Published:** 2026-02-03

**Authors:** Jesse Albano, Allison Mogensen, Cara Christopher

**Affiliations:** 1 Department of Pharmacy, Maimonides Medical Center, Brooklyn, USA; 2 Department of Pharmacy, Yale New Haven Hospital, New Haven, USA; 3 Department of Pharmacy Practice and Administration, Ernest Mario School of Pharmacy, Rutgers, The State University of New Jersey, Piscataway, USA; 4 Department of Pharmacy, Robert Wood Johnson University Hospital, RWJBarnabas Health, New Brunswick, USA

**Keywords:** autoimmune hemolytic anemia, case report, cephalexin, cephalosporins, drug-induced immune hemolytic anemia

## Abstract

Autoimmune hemolytic anemia (AIHA) is an acquired form of hemolytic anemia with several serological subtypes, including drug-induced immune hemolytic anemia (DIIHA). Due to the complex nature of diagnosis, many of the published reports rely on establishing a temporal relationship between drug exposure and symptom onset. To date, only four case reports have suggested a possible association between cephalexin and DIIHA. Here, we present a case of AIHA that occurred following exposure to cephalexin. An 85-year-old female presented with fatigue, dyspnea, and orange urine following initiation of cephalexin three days prior. The patient presented with a positive direct antibody test and a hemoglobin level of 4.7 g/dL, following 15.1 g/dL three days prior. A direct antiglobulin test was initially positive for immunoglobulin G and complement component 3, which eventually tested negative five months later. The patient required blood transfusions, intravenous immunoglobulin, and prednisone with eventual recovery and discharge. The onset of AIHA three days after starting cephalexin and its subsequent resolution following discontinuation of the drug is strongly suggestive of a diagnosis of DIIHA secondary to cephalexin. The patient tolerated other cephalosporins in the past without an immediate immune response. DIIHA is rare, and cephalexin is a seldom-implicated agent. Cephalexin can result in profound DIIHA, requiring transfusions and prolonged hospitalization when used at a typical dose. The case draws awareness to the importance of recognizing signs and symptoms of DIIHA early on so that the offending agent can be discontinued and the appropriate testing and treatment can be performed.

## Introduction

Hemolytic anemia is a condition characterized by abnormal and premature destruction of red blood cells (RBCs). This condition can either be genetically inherited or acquired. Autoimmune hemolytic anemia (AIHA) is an acquired form of hemolytic anemia with several serological subtypes: warm, cold, mixed, paroxysmal cold, and drug-induced immune hemolytic anemia (DIIHA) [1,2]. DIIHA is a rare but potentially life-threatening condition that accounts for approximately 10% of AIHA cases [3].

Diagnosing DIIHA can be challenging due to the need for complex hematologic and serologic laboratory tests. As a result, many of the published reports rely on establishing a temporal relationship between drug exposure and symptom onset. When DIIHA is suspected, pertinent labs that should be assessed include indicators of hemolysis (such as lactate dehydrogenase [LDH], total bilirubin, haptoglobin, and reticulocyte count), a direct antiglobulin test (DAT) with eluate, assessment of complement activation, and testing for the presence of drug-specific antibodies [4]. A positive DAT is the most reliable finding in DIIHA [5].

Over 100 medications have been implicated in DIIHA [6]; however, large-sample studies are extremely limited, and the majority of data is derived from case reports and case series. Penicillin and cephalosporin antibiotics are among the most commonly associated medications with DIIHA [6,7]. A recent study identified a significant association between DIIHA and ceftriaxone, cefixime, and cefpodoxime, but it is unclear if this association extends to the entire cephalosporin class [8].

The exact mechanisms underlying DIIHA are not fully understood, but can be broadly categorized into two subtypes: drug-independent and drug-dependent. In the drug-independent subtype, exposure to a medication triggers the production of autoantibodies that target RBCs both in the presence and absence of the drug, typically leading to mild to moderate hemolysis developing over days to weeks. The drug-dependent subtype involves the binding of the drug and/or its metabolites to RBCs, leading to the formation of drug-specific antibodies. This category can be further divided into drug adsorption and immune-complex mechanisms. The drug adsorption mechanism commonly presents gradually following consistent drug exposure, whereas the immune-complex mechanism can cause severe, acute hemolysis after just one dose of a medication in previously exposed patients [9]. Existing literature has implicated cephalosporins in both drug-dependent and drug-independent pathways [6].

Here, we present a case of AIHA that occurred following exposure to cephalexin with a Naranjo score of three. This score suggests a possible correlation between cephalexin exposure and the development of DIIHA. Patient consent was not obtained due to the retrospective nature of the report, but all patient data has been fully de-identified to protect anonymity.

An abstract summarizing this case was previously published in the proceedings of the 2024 North American Congress of Clinical Toxicology (NACCT) as part of the official post‑meeting abstract collection. The current case report manuscript provides a substantially expanded description, including comprehensive clinical details, diagnostic evaluation, management approach, and patient follow‑up that were not included in the previously published abstract. The full case report has not been published elsewhere and is not under consideration by any other journal. Publication of the meeting abstract does not constitute prior or duplicate case report publication, as it served only to briefly notify the scientific community of this case.

## Case presentation

An 85-year-old Caucasian female weighing 60 kilograms presented to the emergency department (ED) with a complaint of progressive malaise, urinary symptoms, and difficulty ambulating. Past medical history includes type II diabetes mellitus, hypothyroidism, hypertension, and hyperlipidemia. Prior to admission, the patient was taking an extended 28-day course of nitrofurantoin 100 mg by mouth daily and was being followed by urology for recurrent urinary tract infections (UTIs). The nitrofurantoin was discontinued upon ED presentation and throughout admission. Pertinent abnormal lab findings included a platelet level of 108 x 103/µL (normal range: 150-420 x 103/µL), a total bilirubin level of 2.5 mg/dL (normal level: ≤ 1.2 mg/dL), and an aspartate aminotransferase (AST) level of 50 U/L (normal range: 10-35 U/L). A urinalysis was performed, which was concerning for an active UTI, so one dose of ceftriaxone 1 g was intravenously administered and tolerated. The patient was discharged on cephalexin 500 mg by mouth four times daily for 10 days. Of note, the patient had received a three-day course of ceftriaxone eight years prior with a drop in hemoglobin from 13.5 to 11.3 g/dL, without additional signs or symptoms of DIIHA. Table 1 represents the patient's laboratory findings upon presentation.

**Table 1:** Pertinent laboratory findings ED: emergency department

​​Laboratory Parameter, Units	​Patient Value	​Normal Value, Female
​Hemoglobin, g/dL	​5.1, repeat level upon ED arrival of 4.7, and was 15.1 three days prior (see Table 2)	​12-16
​Sodium, mEq/L	​131	​137-145
​Bicarbonate, mEq/L	​19	​22-26
​Blood Urea Nitrogen, mg/dL	​26	​7-21
​Lactate Dehydrogenase, U/L	​494	​135-214
​Aspartate Aminotransferase, U/L	​47	​5-35
​Indirect Total Bilirubin, mg/dL	​2.2	​0.2-1.3
​Direct Bilirubin, mg/dL	​0.6	​< 0.3
​Albumin, g/dL	​3.3	​3.4-5.4
​Platelets, x10^3^/µL	​242	​140-450
​White Blood Cells, x10^9^/µL	​16.4	​4.5-11
​Hematocrit, %	​15	​37-46
​Haptoglobin, mg/dL	​19	​41-165
​Reticulocyte Hemoglobin Content, pg	​42.5	​28-36
​Cobalamin (Vitamin B_12_), pg/mL	​2000	​200-900
​Direct Antibody Test	​3+	​Tested negative 5 months post-ED presentation
​Anti-Immunoglobulin G (IgG)	​3+	
​Anti-Complement C3 (C3)	​1+	
​Panagglutinin	​Positive​	

Notable findings included a rectal temperature of 100.8°F (38.2°C) and hypoxia requiring four liters of oxygen supplementation via nasal cannula (on room air at baseline). Chest radiography was unremarkable. Physical exam demonstrated marked conjunctival pallor; otherwise, the patient was well-appearing.

On day one of admission, intravenous immunoglobulin (IVIG) and oral prednisone 60 mg by mouth daily were initiated, resulting in hemoglobin improvement. The hospital course was complicated by chest pain and shortness of breath during an attempt to transfuse one unit of packed red blood cells in the setting of demand ischemia from profound anemia; however, the patient subsequently received two units of packed red blood cells without complications on day two. On day seven, discharge pharmacotherapy recommendations included prednisone until a stable hemoglobin level greater than 10 g/dL was achieved. Twenty-eight days after hospital discharge, the patient’s hemoglobin was 13.3 g/dL. Immunohematologic evaluation revealed a direct antiglobulin test (DAT) of 3+, with IgG specificity also 3+, and DAT C3 at 1+. Both warm and cold autoantibodies were detected, and panagglutinin was positive, but no alloantibodies were identified. The antibody screen was positive on 6/29, 7/3, and 7/26, but negative on 11/20. Laboratory markers of hemolysis normalized after discontinuation of cephalexin: by 7/26, total bilirubin had resolved to 0.5 mg/dL, LD to 230 U/L, reticulocytes to 2.0%, and haptoglobin to 106 mg/dL. DAT C3 was negative, and DAT positivity was reduced, further supporting the diagnosis of drug-induced immune hemolytic anemia. Table 2 represents the hemoglobin results throughout the hospital stay.

**Table 2:** Change in hemoglobin during clinical course IV: intravenous; IVIG: intravenous immunoglobulin; PRBCs: packed red blood cells

Day	Hemoglobin (g/dL)	Therapeutic Interventions
-3 (ED Visit 1)	15.1	Nitrofurantoin was discontinued and ceftriaxone 1 g IV was administered once
-2	-	Cephalexin 500 mg four times daily initiated
0 (ED Visit 2)	4.7	Cephalexin discontinued
1	4.6 (morning collection) 6.5 (evening collection)	IVIG and prednisone initiated
2	7.4	Transfusion of two units of PRBCs
3	7.0	
4	7.4	IVIG continued through day five
5	8.7	
6	8.9	
7 (Discharged)	9.0	Prednisone continued on discharge
28	13.3	

## Discussion

DIIHA is a challenging and often overlooked diagnosis, frequently relying on establishing a temporal association between drug administration and the onset of clinical symptoms and laboratory findings. This is particularly true for DIIHA associated with cephalexin, as supporting literature for this link is scarce. To date, only four case reports have suggested a possible association between cephalexin and drug-induced hemolytic anemia (DIHA) (Table 3) [10-13]. Three of these case reports demonstrate an immune-mediated reaction [11-13], while the case report by Thiessen and Kraleti describes a case of non-immune-mediated hemolytic anemia [10].

**Table 3:** Characteristics of patients with suspected DIIHA to cephalexin DAT: direct antiglobulin test; DIIHA: drug-induced immune hemolytic anemia; UTI: urinary tract infection

Reference	Forbes (1972) [13]	Manoharan (1987) [12]	Baradhi (2015) [11]	Thiessen (2017) [10]	Our Patient
Sex/age	M/14 y	F/23 y	F/61 y	F/44 y	F/85 y
Diagnosis	Pneumonitis	Bronchiectasis prophylaxis	Bronchitis	UTI	UTI
Cephalexin regimen	2 g daily	500 mg PO four times daily	Unknown	Unknown	500 mg PO four times daily
Interval between reaction and first drug administration	9 days	4 months	10 days	6 days	2 days
DAT	Positive	Positive	Positive	Negative	3+
Anti-IgG	Negative	Strongly positive	Positive	Negative	3+
Anti-C3	Negative	Strongly positive	Positive	Negative	1+
Hemoglobin nadir (g/dL)	6	5.7	5.5	5.7	4.6
Reticulocyte peak (%)	13	4	4	4.21	>18
Haptoglobin nadir (mg/dL)	Unknown	20	38	<30	<10

In our patient, laboratory findings of hemolytic anemia in conjunction with a positive DAT for both IgG and C3 are consistent with AIHA. Following the discontinuation of cephalexin, the hemolytic process resolved, and AIHA workup results progressively normalized. The onset of AIHA three days after starting cephalexin and its subsequent resolution following discontinuation of the drug is strongly suggestive of a diagnosis of DIIHA secondary to cephalexin, further supported by a Naranjo score of three [14].

As with many cases of DIIHA, several factors complicate diagnostic certainty. Notably, our patient received a single dose of ceftriaxone immediately before starting cephalexin. Although ceftriaxone is more commonly associated with DIIHA, multiple factors make it a less likely cause in this case and instead support cephalexin as the probable agent [6,15]. These include the delayed onset of hemolysis and the fact that ceftriaxone had been discontinued prior to the major hemolytic episode. 

First, our patient had previously tolerated a three-day course of ceftriaxone (1 g daily) without obvious reactions. The literature on ceftriaxone-induced DIIHA typically describes either a rapid time to reaction, often observed in pediatric patients, or a delayed reaction following repeated or high-dose exposure over days to weeks [15-17]. If our patient had developed ceftriaxone-dependent antibodies during their initial course of ceftriaxone, a more immediate immune response and subsequent hemolysis would be expected upon re-exposure rather than the delayed reaction observed in this case. Otherwise, a single dose of ceftriaxone triggering such a profound but delayed immune response would be atypical based on the existing literature [15-17]. While this does not entirely exclude the possibility of ceftriaxone as the culprit, re-exposure or continued treatment is a key factor in the development of drug-dependent antibodies [18]. The sustained course of cephalexin, taken four times daily over several days, aligns more consistently with this pattern. 

Second, our patient had previously received separate, seven-day courses of both cefdinir and cefuroxime a year prior without an obvious reaction. Although cross-reactivity among cephalosporins is rare, when it does occur, it is due to similarities in the R1 or R2 side chain structure [19]. Both cefuroxime and cefdinir share structural similarities with ceftriaxone in their R1 side chains, and several cases of positive cefuroxime skin tests have been reported in patients with a documented ceftriaxone allergy [19]. In contrast, cephalexin does not share any such structural similarities with these antibiotics. While minor differences in side-chain structure can lead to significant variations in antigenicity, shared structural features between the metabolites of ceftriaxone and structurally similar cephalosporins result in cross-reactivity of drug-dependent antibodies [18,20]. This suggests that a similar immune response should be observed following exposure to structurally similar cephalosporins. Given that our patient tolerated full courses of both cefdinir and cefuroxime, this further supports the likelihood of cephalexin being the causative agent in this case of DIIHA.

A limitation of our case is the lack of a test for the presence of cephalexin-dependent antibodies, which would provide definitive confirmation of cephalexin as the offending agent. Unfortunately, a lab test to assess drug-dependent antibodies was not performed in this case. However, the presence of warm autoantibodies at presentation, which resolved after several months, along with a negative finding for alloantibodies, strongly suggests a drug-induced etiology.

It is important to note that drug-dependent antibodies can mimic autoantibodies, especially when the test is conducted while drug-antibody complexes are still in circulation [5]. In our case, the patient’s antibody status remained positive for at least one month, exceeding the time expected for cephalexin to clear the blood. This finding raises the possibility of a drug-independent antibody response, making the presentation indistinguishable from idiopathic AIHA.

Therapy for DIIHA involves stopping the offending agent immediately. Supportive care with transfusion of red blood cells is advised if necessary. Additional treatment options include steroids and IVIG [3,5]. Steroids have only demonstrated benefit in drug-independent DIIHA, as autoantibodies are present. In such cases, IVIG has also shown some success [5]. Treatment outcomes are challenging to interpret, however, as therapy often coincides with the cessation of the offending agent, clouding the judgment of how impactful each intervention was individually. In this case, blood transfusion, steroids, and IVIG were all administered, with improvement leading to discharge in eight days and improvement of laboratory markers of hemolysis on follow-up three weeks thereafter.

## Conclusions

Our case demonstrates that cephalexin can result in profound DIIHA requiring blood transfusions and prolonged hospitalization when used at a typical dose. The case raises awareness of the importance of recognizing the signs and symptoms of DIIHA early on so that the offending agent can be rapidly discontinued and appropriate testing and monitoring can be performed.
